# Isoniazid causing pleural effusion

**DOI:** 10.4103/0253-7613.41045

**Published:** 2008

**Authors:** S.K. Singh, Z. Ahmad, D.K. Pandey, V. Gupta, S. Naaz

**Affiliations:** Department of Tuberculosis and Respiratory Diseases, JN Medical College, AMU, Aligarh, UP, India; 1 Department of Medicine, JN Medical College, AMU, Aligarh, UP, India

**Keywords:** Antitubercular drug, isoniazid, paradoxical response, pleural effusion

## Abstract

Isoniazid (INH) is a first-line antitubercular drug. We report a case of a patient who developed a pleural effusion 2 months after starting antitubercular treatment for spinal tuberculosis. Isoniazid was found to be the culprit and its discontinuation caused subsidence of the effusion.

## Introduction

Paradoxical response to chemotherapy in tuberculosis is the worsening of a preexisting lesion or the appearance of a new lesion during antitubercular treatment. Such lesions usually disappear on their own but sometimes the administration of steroids or the withdrawal of the offending drug is required.

## Case History

A 24-year-old male presented to our outpatient department with complaints of fever, chest pain, and shortness of breath of 1 week's duration. His clinical examination showed a pulse rate of 90/min, respiratory rate of 20/min, and blood pressure of 110/80 mmHg. He was anemic. Cyanosis and icterus were absent. He had history of spinal tuberculosis for which antitubercular drugs (rifampicin, isoniazid [INH], pyrazinamide, and ethambutol) had been started 2 months earlier. Examination of the respiratory system revealed decrease in chest movements on the right side. On percussion, there was stony dullness on the right side, and the breath sounds were absent on auscultation of the lower lung fields. The left side of the chest was normal.

**Figure 1:**
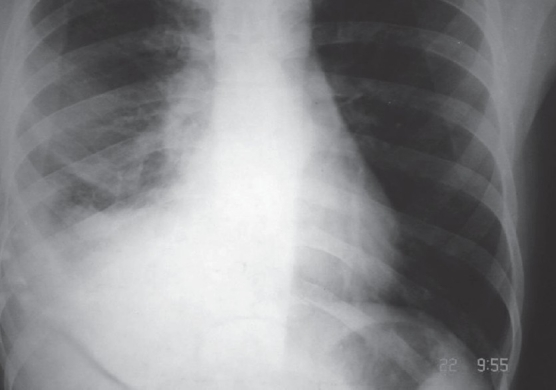
X-ray chest showing right-sided effusion and pneumonitis

Laboratory investigation showed hemoglobin of 9 gm%; the total leucocyte count was 9600/cu mm, with 74% neutrophils, 22% lymphocytes, and 4% eosinophils. Renal and liver function tests were within normal limits. Test for HIV was negative. Sputum smear was negative for *Mycobacterium tuberculosis*. The chest X-ray (PA view) showed a right-sided pneumonitis with effusion [Figure 1], although there was no evidence of pleuroparenchymal tuberculosis in a chest X-ray that had been taken earlier. MRI of the spine was done and, when compared with the MRI that had been done earlier, it showed improvement in the spinal lesion.

On thoracocentesis there was an exudative effusion; cytology of the fluid revealed lymphocytes - 90%; neutrophils - 4%, and mesothelial cells - 6%. LE cells were not found in the pleural fluid and the ADA level was not elevated (12 IU/l). Antinuclear antibody (ANA) titer in the fluid was elevated (1:320).

**Figure 2:**
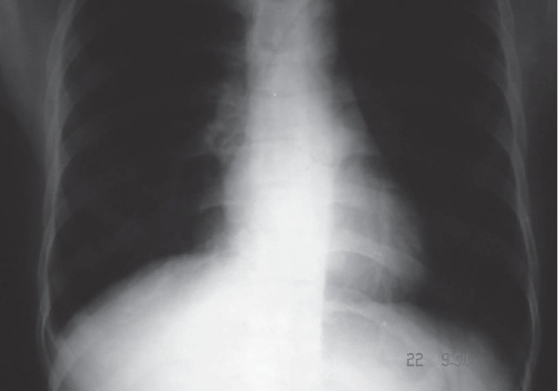
X-ray chest showing improvement in effusion after withdrawing of INH

We advised continuation of the antitubercular treatment and added a corticosteroid to his drug regimen; however, the signs and symptoms of the effusion persisted. Considering the possibility of an INH-induced effusion, INH was stopped, upon which the patient started showing clinical improvement. A repeat chest x-ray, taken 2 weeks after stopping INH, showed disappearance of the pleural effusion [Figure 2].

## Discussion

INH is one of the first-line drugs for the treatment of tuberculosis. It is a bactericidal drug. After absorption, it is distributed to all body organs. The mechanism of action is the inhibition of mycolic acid synthesis. INH is metabolized in the liver by acetylation and is excreted in urine. The various untoward effects of INH include epigastric discomfort, neurological manifestations, hepatitis, etc. These effects are dose related. The lupus phenomenon, which has also been reported with INH, is an idiosyncratic reaction.[1] There are only a few cases of INH-induced pleural effusion described in literature.[2] INH-induced effusion usually begins 3-12 weeks after starting chemotherapy and regresses after change of therapy or introduction of steroids or both.[2] Although many theories have been put forward to explain the phenomenon, such as immunological rebound[3] or an interaction between mycobacterial products and improving host immunity,[4] none of these theories have been confirmed.

In our case, the elevated ANA titer was suggestive of INH-induced lupus; however, the test for LE cells was negative and there was no improvement in symptoms after the introduction of a steroid into the treatment. The patient showed improvement only after discontinuation of INH, ie, dechallenge was positive. We, however, did not try reintroduction of INH.

There are increasing reports of cases of drug resistance, but the appearance of new lesions, such as an effusion or a pneumonitis, in a patient on treatment for tuberculosis may also be drug-induced; this possibility must always be kept in mind. Paradoxical worsening of the lesion can also occur in cerebral tuberculoma and tubercular lymphadenitis.[4] In our patient, the aspirated pleural fluid was not characteristic of tuberculosis, which suggested that this was not a case of drug-resistant tuberculosis.
